# Screening for Autism Spectrum Disorder in Toddlers During the 18- and 24-Month Well-Child Visits

**DOI:** 10.3389/fpsyt.2022.879625

**Published:** 2022-04-26

**Authors:** Ying Zhang, Zhaoe Zhou, Qiong Xu, Huiping Li, Yujing Lv, Guowei Zhu, Ping Dong, Dongyun Li, Yi Wang, Xinrui Tang, Xiu Xu

**Affiliations:** ^1^Department of Child Healthcare, Children's Hospital of Fudan University, Shanghai, China; ^2^Department of Child Healthcare, Xuhui Maternal and Child Healthcare Hospital, Shanghai, China

**Keywords:** autism spectrum disorder, early screening, M-CHAT-R/F, BOT, community-based

## Abstract

**Objective:**

Early screening contributes to the early detection of children with autism spectrum disorder (ASD). We conducted a longitudinal ASD screening study in a large community setting. The study was designed to investigate the diagnostic rate of ASD screening and determine the effectiveness of ASD screening model in a community-based sample.

**Methods:**

We enrolled children who attended 18- and 24-month well-child care visits in Shanghai Xuhui District. Modified Checklist for Autism in Toddlers, Revised with Follow-up (M-CHAT-R/F) and Binomial Observation Test (BOT) were selected as screening instruments. Screen-positive children were referred to a tertiary diagnostic center for comprehensive ASD diagnostic evaluation. Screen-negative children received well-child checkups and follow-up every 3–6 months until age three and were referred if they were suspected of having ASD.

**Results:**

A total of 11,190 toddlers were screened, and 36 screen-positive toddlers were diagnosed with ASD. The mean age at diagnosis for these children was 23.1 ± 4.55 months, diagnosed 20 months earlier than ASD children not screened. The diagnostic rate of ASD was 0.32% (95% CI: 0.23–0.45%) in this community-based sample. In addition, 12 screen-negative children were diagnosed with ASD during subsequent well-child visit and follow-up. The average diagnostic rate of ASD rose to 0.43% (95% CI: 0.32–0.57%) when toddlers were followed up to 3 years old. The positive predictive values (PPVs) of M-CHAT-R/F, M-CHAT-R high risk, and BOT for ASD were 0.31, 0.43, and 0.38 respectively.

**Conclusion:**

Our findings provide reliable data for estimating the rate of ASD detection and identifying the validity of community-based screening model. M-CHAT-R/F combined with BOT can be an effective tool for early detection of ASD. This community-based screening model is worth replicating.

## Introduction

Autism spectrum disorder (ASD) is a group of heterogeneous neurodevelopmental disorders, which are characterized by deficits in social communication and interaction, and restricted and repetitive patterns of behaviors, interests, or activities (1). The current prevalence is assessed to be about 1.5% in developed countries and 1% in worldwide (2, 3). In China, the prevalence of ASD has been reported ranged from a low of 0.2% to as high as 1% (4, 5). Zhou et al. reported a prevalence of 0.7% among 6- to 12-year-old children in 2019 (5), which is the largest epidemiological study in China to date. ASD tends to be accompanied by a kind of serious neuropsychiatric disorder in adulthood if there is no effective intervention in time, which might become a heavy burden to an individual, a family, or even the whole society (6–8). Studies have shown that early behavioral treatment can largely improve the cognitive and adaptive abilities of children with ASD, and early intensive interventions before age three can improve the prognosis to a large extent (9–11).

Early screening and early diagnosis play a key role in affecting the prognosis of this disease. Signs of ASD can occur very early, even in the first year of life and a diagnosis can be made at as early as 12 months (12). A formal diagnosis may be possibly made only at 18–24 months of age, and the stability of the diagnosis is quite high over time (13, 14). Therefore, early diagnosis of ASD is possible. However, at present, the diagnosis of ASD is made around the age of 4–5 years on average (15–17). There is a significant delay between the onset of ASD symptoms and diagnosis, which means that young children miss the opportunity for intervention during the optimal period of neuroplasticity. The American Academy of Pediatrics (AAP) recommends that children be screened for ASD at the 18- and 24-month checkups (18). There are many studies of early screening for ASD in developed countries (19–24). In a large early screening study, Robin et al. reported a diagnostic rate of 0.67% in toddlers (19). In another screening study of low-risk young children, the diagnostic rate of ASD was 0.65% (20). The initiative of early ASD screening starts much later in China than that in the developed countries. The first large early screening study for ASD in China was initiated by our team in 2013 and lasted for 4 years. In that study, the early diagnostic rate of ASD was 0.21% (25). With the large population in China, the early screening will detect many children with ASD. Early screening would significantly shorten the average time from onset to diagnosis and intervention of these children, thus increasing the possibility of improving their prognosis and relieving the families' burdens.

For widespread use and well-implementation, screening tools should be brief, easy to complete and effective. The Modified Checklist for Autism in Toddlers, Revised with Follow-up (M-CHAT-R/F) is a two-stage screening tool for ASD that has been reported to have adequate sensitivity and specificity (19, 20). The M-CHAT-R/F has been translated into more than 40 languages and requires little time and cost, making it one of the most widely used ASD screening tools. However, few studies have evaluated whether this screening tool performs adequately in Chinese Han toddlers. Similar to other screening questionnaires, the screening results are closely related to the quality of parental completion of a symptom checklist (19, 26). If parents underreport their children's symptoms, there will be a possibility of misdiagnosis. Brief observation of physicians who administer screening may increase the likelihood of early identification (25). We speculate that M-CHAT-R/F combined with brief observation of physicians would be an effective screening tool.

This study aimed to investigate the diagnostic rate of ASD screening and identify the validity of screening model combining M-CHAT-R/F with brief observation of physicians in the current Chinese three-level healthcare system.

## Methods

### Participants

This study was implemented at 13 community healthcare centers in Xuhui District, Xuhui Maternal and Child Healthcare Hospital (XMCHH), and Children's Hospital of Fudan University (CHFU). Xuhui District, a central district of Shanghai with about 5,000–6,000 births per year, has a well-established three-level child healthcare system. Toddlers aged 18 to 24 months who were at well-child visit in Xuhui district from January 2018 to December 2019 were enrolled. Parents of all participants provided informed consent. The study was approved by the Ethical Committee of the Children's Hospital of Fudan University.

### Screening Instruments and Procedure

When children aged 18–24 months attended routine well-child visits at community healthcare center, they underwent early screening using the M-CHAT-R/F and Binomial Observation Test (BOT).

**Figure 1:**
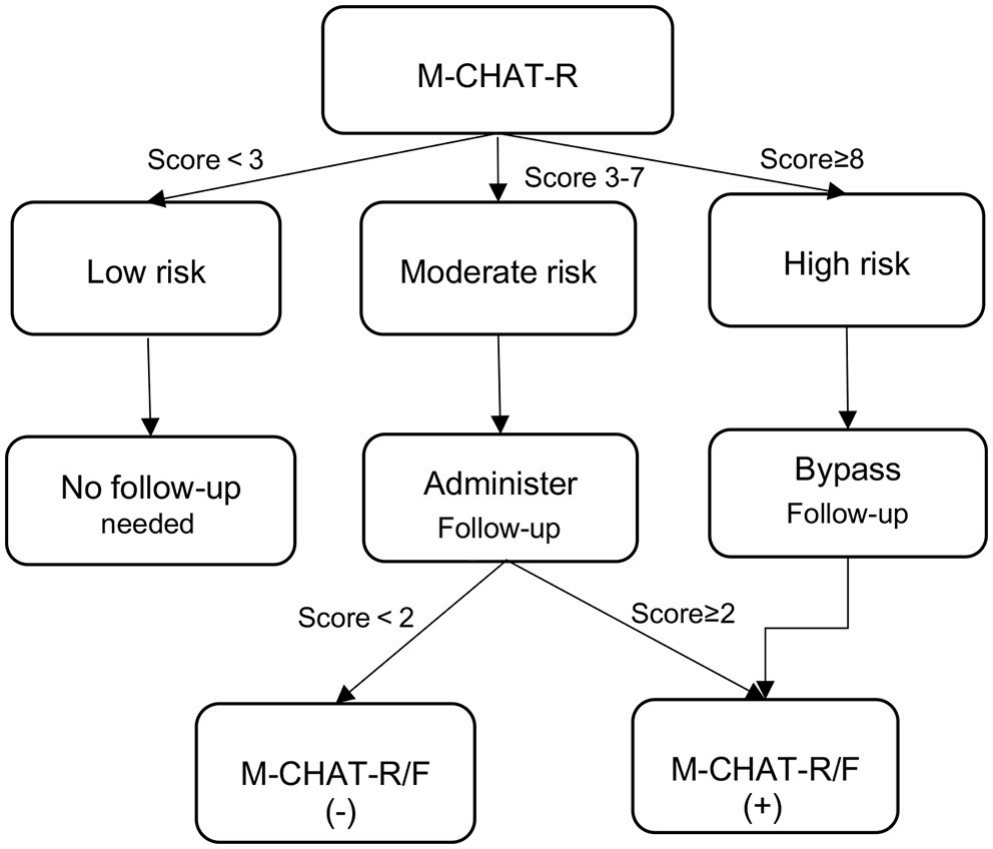
Recommended algorithm based on 2-stage M-CHAT-R/F screening.

M-CHAT-R/F is a two-stage screening questionnaire consisting of 20 questions on a scale of 0–20 (www.mchatscreen.com). The M-CHAT-R refers to the initial screening, while the M-CHAT-R/F refers to the second-stage screening process with follow-up. Positive screening for the M-CHAT-R includes 3 or more high-risk responses (total score: 3–7, moderate risk; total score: 8–20, high risk). If children receive a score of “high risk” (total score ≥ 8) on M-CHAT-R, they would bypass the follow-up and are considered positive on M-CHAT-R/F. If with a score of “moderate risk” (total score: 3–7), a screening process for follow-up is required. Positive screening for follow-up (M-CHAT-R/F) includes 2 or more risky reactions (Figure 1). In this study, parents first completed 20 questions in M-CHAT-R according to their children's daily performance. If children received a score of “moderate risk”, the follow-up interview was completed by the primary care physicians (PCPs) at the 13 community healthcare centers.

**Figure 2:**
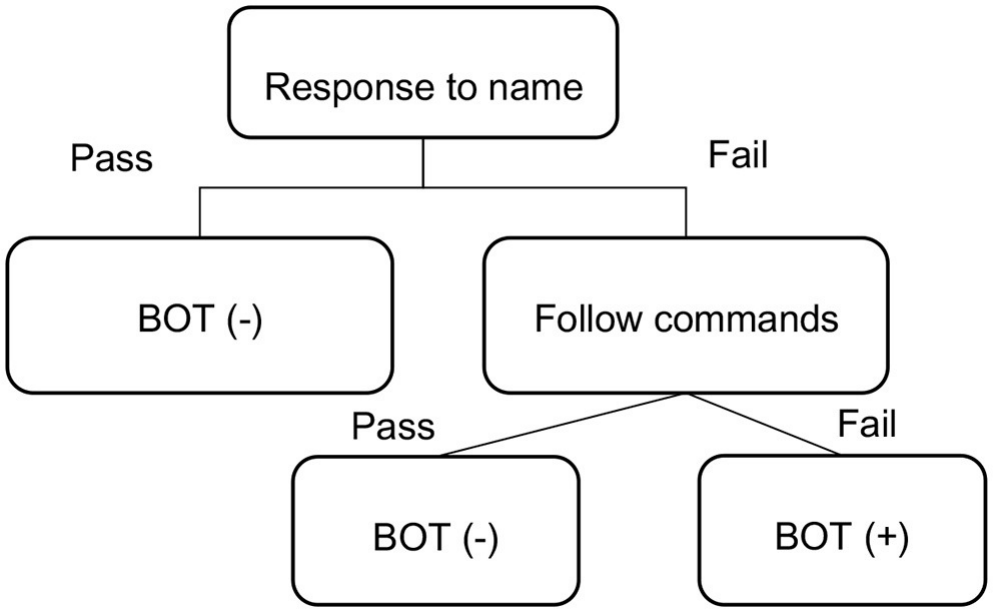
Procedure of the binomial observational test (BOT).

Besides M-CHAT-R/F, PCPs also administered a two-step observational test which was called Binomial Observation Test (BOT). The first step is “Response to name” and the second step is “Follow commands.” In the first step, PCP called the child's name twice in a clear voice at a normal volume. If the child failed to look toward the PCP, the second step was performed. In the “Follow commands” step, the child was required to follow two simple instructions such as waving goodbye, or blowing a kiss. If the child could not follow either command, she/he failed the test. It meant that the child was screened positive on BOT (Figure 2).

Although the whole screening process was completed by PCPs at the 13 community healthcare centers, the pediatricians at XMCHH were responsible for supervision and quality management of PCPs' screening work.

### Referral and Diagnosis

Toddlers who screened positive on M-CHAT-R/F and/or BOT were deemed positive and were referred to the tertiary diagnostic center: CHFU, for comprehensive ASD diagnostic evaluation and counseling on next step recommendations. The timing of these referrals was a hinge, as it could directly impact the age of diagnosis as well as the receipt of appropriate interventions. Therefore, a special green channel had been set up to help get referrals as soon as possible.

The diagnosis of ASD was made by developmental pediatricians at CHFU based on the ASD criteria in the Diagnostic and Statistical Manual of Mental Disorders, Fifth Edition (DSM-5), and further confirmed by the Autism Diagnostic Observation Schedule, second edition (ADOS-2). When parents refused to complete evaluations, a telephone follow-up was completed by pediatricians at CHFU and XMCHH.

To maximize the detection of missed cases, children who screened negative would take well-child checkups and follow-up at community healthcare centers every 3–6 months until they reached 3 years of age. Children were also referred to the CHFU if they were suspected of having ASD by PCPs during follow-up well-child visit.

### Statistical Analyses

R statistical software was used to perform data analysis. Measurements such as ages were presented as mean ± SD (X ± S), and numerical data such as number of patients were presented as numbers and percentages. The *t*-test was applied for detecting differences in measurement data between groups, and the Chi-Square test was used to analyze differences in numerical data. If *P* < 0.05, it was considered statistically significant for all tests. Also, 95% Confidence interval (CI) were determined on the basis of the approximate normal distribution method.

## Results

### M-CHAT-R/F Screening and Diagnostic Outcome

From January 2018 to December 2019, there were 11,190 toddlers with 18- and 24-month well-child visit were screened in Xuhui District. Of the total 11,190 toddlers, 474 (4.2%) were positive on M-CHAT-R or BOT and 126 (1.1%) were positive on M-CHAT-R/F or BOT. Ultimately, 36 children were diagnosed as ASD. The diagnostic rate of ASD through community screening was 0.32% (95% CI: 0.23–0.45%).

### M-CHAT-R

Among the 474 children with positive screening results, 459 children had positive M-CHAT-R screening results (402 toddlers screened positive on M-CHAT-R only and 57 toddlers screened both positive on M-CHAT-R and BOT), with a screen-positive rate of 4.1% (459/11,190). Among the 459 children, 33 children were finally diagnosed with ASD, and the positive predictive value (PPV) of M-CHAT-R for ASD was 0.07 (33/459). Forty-six children scored in the high-risk range on M-CHAT-R and 20 of them were diagnosed with ASD. The PPV of M-CHAT-R high risk for ASD was 0.43 (20/46).

### M-CHAT-R/F

The follow-up interviews were administered by trained PCPs from community healthcare centers. Among the 459 toddlers who were positive on M-CHAT-R, 22.9% (105) of them were screened positive on M-CHAT-R/F. Of the 105 children, 33 children were diagnosed with ASD. The PPV of M-CHAT-R/F was 0.31 (33/105), which was significantly higher than M-CHAT-R (χ^2^ = 46.271, *P* < 0.001). With a comparison between PPV of M-CHAT-R high-risk and that of M-CHAT-R/F for ASD, it is found that the former is higher with no statistically significant difference (χ^2^ = 1.5441, *P* = 0.214).

### BOT Screening and Diagnostic Outcome

A total of 72 toddlers were screened positive on BOT (15 toddlers were screened positive on BOT only and 57 toddlers were screened both positive on M-CHAT-R and BOT). Of the 72 toddlers, 27 children were finally diagnosed as ASD, and the PPV of BOT for ASD was 0.38 (27/72), which was significantly higher than M-CHAT-R (χ^2^ = 54.065, *P* < 0.001) and similar to M-CHAT-R/F (χ^2^ = 0.45782, *P* = 0.4986). There were 57 toddlers screened both positive on M-CHAT-R and BOT. Of these 57 children, 24 were finally diagnosed with ASD. The PPV of M-CHAT-R & BOT was 0.42 (24/57). See Table 1 for PPVs of M-CHAT-R, M-CHAT-R high risk, M-CHAT-R/F, and BOT.

**Table 1:** PPVs of M-CHAT-R, M-CHAT-R/F, and BOT.

	**Total number**	**Diagnosed with ASD**	**PPV**
M-CHAT-R (+)	459	33	0.07
M-CHAT-R high risk	46	20	0.43
BOT (+)	72	27	0.38
BOT & M-CHAT-R (+)	57	24	0.42
M-CHAT-R/F (+)	105	33	0.31

**Figure 3:**
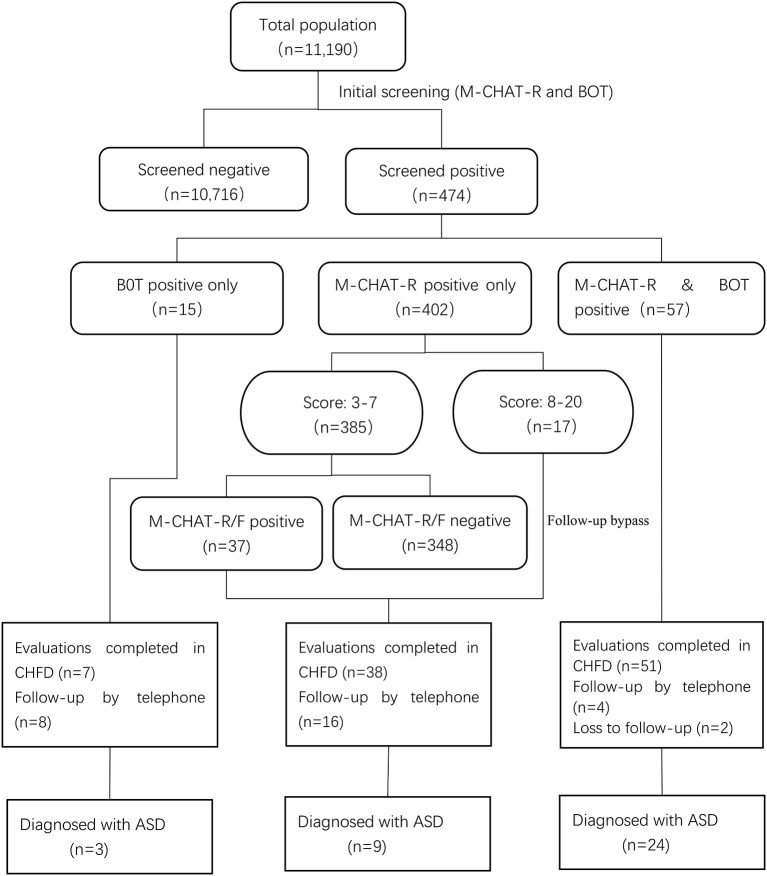
Flowchart displaying the screening and diagnosis results for all toddlers studied.

In addition, there were 15 children who were positive on BOT but negative on M-CHAT-R. Of these 15 children, 3 were diagnosed with ASD. It meant that there was a 20% chance of being diagnosed with ASD in these children. They accounted for 8.3% (3/36) of all screen-positive ASD patients. We can see the flowchart and screening results in Figure 3.

### Comparison to the Non-screened ASD Group in Shanghai

Thirty-six screen-positive children were diagnosed with ASD on the community-based early ASD screening model. Among them, 30 were male, and 6 were female (sex ratio = 5:1). The mean age at screening and diagnosis were 21.1 ± 2.71 months and 23.1 ± 4.55 months, respectively. The average interval time from initial screening to diagnosis was 2 months.

We compared the diagnostic age of community screened children with those who located in Shanghai but didn't receive community ASD screening. During the same period, 473 ASD children located in Shanghai were first diagnosed without ASD screening, including 399 males and 74 females. The ratio of males to females in non-screened group was 5.4:1, which was similar to the community screened ASD group. Overall, 60.3% of children with ASD did not have a comprehensive evaluation until after age 3 years old. The average diagnostic age of these patients was 43.2 ± 17.91 months. The diagnostic age of community screened group was significantly younger than the non-screened ASD group in Shanghai (*P* < 0.001). On average, screen-positive children were diagnosed 20 months earlier than children not screened (see Table 2).

**Table 2:** Comparison of sex distribution and age at diagnosis between community screening ASD group and non-screening ASD group.

	**Total number**	**Age at diagnosis** **(x ± s)**	**Male/female**
Community screening	36	23.1 ± 4.55	5:1
Non-screening group	473	43.2 ± 17.91	5.4:1
χ 2/t		−17.507	2.43*10^−30^
*P*-value		<0.0001	0.9999

### False Negative Cases

A total of 12 children with negative screening results were diagnosed with ASD at the age of 30 ± 4.1 months, diagnosed 13 months earlier than children not screened on average. These 12 children all had negative screening results at 18 months of age and were referred to CHFU because of the PCPs' concern about ASD at the age of 27 ± 3.4 months. The interval time from initial negative screening to diagnosis was 12 ± 4.2 months.

### Total Diagnostic Rate of ASD

Thirty-six screen-positive children were diagnosed with ASD, and 12 screen-negative children were diagnosed with ASD during follow-up well-child visit at community healthcare centers. Totally, 48 children were diagnosed with ASD.

In addition, 30 children who screened positive on M-CHAT-R/F or BOT refused to take an evaluation at CHFU. Among the 30 children, 16 children were positive on M-CHAT-R/F only, 8 children were positive on BOT only, and 6 children were M-CHAT-R/F and BOT both positive. The follow-up telephone interviews were made with their parents. The primary reason for refusal of evaluation was that the parents did not believe the child had an ASD-related problem and reported as social normal (28/30, 93.3%). And the secondary reason was that the parents moved house and could not be contacted (2/30, 6.7%). The 30 missing data were imputed as non-ASD, and an adjusted diagnostic rate was calculated. The 95% CI for the diagnostic rate was determined based on the approximate normal distribution method. When children aged 18–24 months were followed up to 3 years of age at community healthcare center, the average diagnostic rate of ASD was 0.43% (48/11,190, 95% CI: 0.32–0.57%).

## Discussion

Shanghai, one of the largest cities in China, has a well-established three-level child healthcare system that provides basic healthcare services and monitoring for children aged 0–3 years. PCPs at the community healthcare center provide screening, referral (level 1); pediatricians at the district maternal and child health centers provide monitoring, further referral (level 2); and pediatricians at the specialized children's hospitals provide diagnosis, consultation, and treatment (level 3). The three-level child healthcare system plays the significant role of pediatricians at each level. In particular, the PCPs at the community healthcare center, as the front line of defense, are essential. The three-level connection and cooperation can achieve early screening, early diagnosis and early intervention for ASD.

The AAP recommended screening for ASD at 18 and 24 months in 2007 (18). In 2016, the US Preventive Services Task Force (USPSTF) published a controversial report concluding that there was insufficient evidence to assess the balance of benefits and harms of early ASD screening (27). In response, the AAP promptly issued a statement on their website, remaining committed to their recommendation for universal screening of 18- and 24-month-old children for ASD. Some other professional and advocacy organizations such as the American Academy of Child Neurology, the American Academy of Child and Adolescent Psychiatry and the American Academy of Pediatrics' Bright Futures also recommend early universal screening (28). There is ample evidence that strongly supports the universal ASD screening in children aged 18–24 months (29, 30). In 2017, the Chinese expert consensus on early screening for ASD, issued by the Chinese Medical Association (CMA), recommended that pediatricians at all levels of hospitals should provide regular early ASD screening for infants and toddlers at 9, 18, and 24 months of age, on the basis of China's three-level child healthcare system (31). The corresponding author of the present paper is also one of the main contributors to this expert consensus.

The current study was based on the three-level child healthcare system. However, the whole screening process was completed in community (level 1). Children who screened positive at community healthcare center (level 1) were directly referred to the tertiary diagnostic center (level 3) for diagnostic evaluation. What pediatricians at the district maternal and child healthcare hospital (level 2) should do is playing a connecting role in supervision and quality management of the community healthcare center. Such a referral model that lessens intermediate referral can reduce the loss of follow-up visit and avoid the potential time delay.

In our previous study, only 64.8% (283/437) of positive cases from primary screening (level 1) completed the face-to-face second screening (level 2) (25). Through an analysis of causes of loss of follow-up visit during the intermediate referral from the primary to the secondary, we found that many families skipped the secondary hospital due to the inconvenient transportation or the urgent demands for medical treatment. Hence, we canceled the intermediate referral from the community healthcare center to the district maternal and child healthcare hospital in this longitudinal study.

In this study, a total of 11,190 toddlers were screened and 36 screen-positive children were diagnosed with ASD. Screen-positive children were diagnosed 20 months earlier than children not screened, which means they could significantly improve the long-term outcomes. The diagnostic rate of ASD through community screening was 0.32% (95% CI: 0.23–0.45%), which was higher than the 0.21% we reported in 2018 (25). There were several explanations that may account for the increased early detection rate. First, the next follow-up screening was completed immediately after the initial M-CHAT-R screening, which reduced the loss of follow-up visits. Second, the experience of PCPs increased. PCPs who started ASD screening back in 2013 had gained some experience. They were more agile than before to detect toddlers with ASD. Third, public awareness of ASD had increased. In recent years, the extensive scientific knowledge propagation of ASD by the government and media had caught more attention among the public. Thus, parents were more likely to detect abnormal behaviors of their children and were willing to send them to the tertiary hospital for diagnosis. However, this rate was still a little lower than studies conducted in some developed countries (19–22). This may be due to different study designs, the ethnic and geographical differences.

To minimize missed false-negative cases, children who screened negative at 18–24 months received routine well-child checkups and follow-up every 3–6 months at the community healthcare centers until they reached 36 months of age. If they were suspected of having ASD by PCPs, they would be referred to the CHFU for evaluation and diagnosis. Benefiting from the healthcare and referral networks, a total of 12 children with negative screening results at 18 months of age were identified as having ASD. They were diagnosed with ASD at the mean age of 30 months, 13 months earlier than the children who did not go through community screening and systematic management. We recommend that children who pass ASD screening at 18 months still need developmental surveillance until at least 3 years old. The follow-up well-child visit at community healthcare center and the referral network are necessary.

The diagnostic rate of ASD was 0.43% (95% CI: 0.32–0.57%) When children were followed up to 3 years of age at the community healthcare center. This rate was slightly lower than the prevalence of ASD in children aged 6–12 years reported by Zhou et al., which was 0.7% (5). In any case, it cannot be expected that all cases of ASD will be found in toddlers. In fact, children with high-functioning autism are usually diagnosed at preschool or even school age, when they enter a group setting with high demands on social communication. We investigated the sex ratio in screen-positive ASD and non-screened ASD groups, which was very similar in both groups, around 5:1 (male/female). Interesting, the sex ratio in false-negative cases was also 5:1 (male/female).

The PPV of M-CHAT-R/F was 0.31, which was significantly higher than M-CHAT-R. The follow-up interview can improve PPV and conserve evaluative resources. Therefore, it is necessary to administer the follow-up interviews for children with medium risk on M-CHAT-R screening. The PPV of M-CHAT-R/F for ASD reported in previous studies was between 0.4 and 0.5 (17, 18), which was slightly higher than 0.31 in this study. However, even without follow-up interviews, the PPV of M-CHAT-R high risk for ASD was 0.43, which was still higher than M-CHAT-R/F and showed good diagnostic significance. Therefore, we recommend that children who receive score of “high risk” on M-CHAT-R can skip follow-up interviews and should receive immediate specialist evaluation.

There were 15 children who were positive on BOT but negative in the questionnaire. Of these 15 children, 3 were diagnosed with ASD, accounted for 8.3% of all screen-positive ASD toddlers. The PPV of BOT was 0.38, which was slightly higher than M-CHAT-R/F (0.31). BOT is two-step behavioral test and very easy to practice. The inclusion of BOT in the screening process can reduce missed diagnoses and affect the prognosis of a small number of toddlers with ASD. Moreover, it is helpful to improve the early identification skills of the PCPs by training and test through the simple process of observation, thus fully exerting themself to the front line of defense.

The major limitation of this study is that it was carried out in Shanghai Xuhui District, bringing about certain regional limitations. Currently, similar research is urgently needed in other areas. We have planned multiple studies in other jurisdictions to get more robust data fur further analysis. These studies are still ongoing and we can look forward to the release of the results in the future.

## Conclusions

Based on the above results, we can conclude that an efficient large-scale ASD screening in a large community-based population need the support from a well-established child healthcare system, primary care physicians with basic knowledge of ASD screening, and a standardized screening tool. In China, child healthcare system is well-established in most of the cities. We can rely on the three-level child healthcare system, reliable screening tools and surveillance strategies to conduct ASD screening in community-based populations. The screening model combining M-CHAT-R/F with BOT is worth replicating. With a large population in China, a considerable number of ASD cases can be detected and their families will benefit from early ASD screening.

## Data Availability Statement

The raw data supporting the conclusions of this article will be made available by the authors, without undue reservation.

## Ethics Statement

The studies involving human participants were reviewed and approved by the Ethical Committee of the Children's Hospital of Fudan University. Written informed consent to participate in this study was provided by the participants' legal guardian/next of kin.

## Author Contributions

All authors listed have made a substantial, direct, and intellectual contribution to the work and approved it for publication.

## Funding

This study was supported in part by the Key Subject Construction Project of Shanghai Municipal Health Commission (shslczdzk02903), the National Natural Science Foundation of China (NSFC, 61733011), and Industry-University-Research High Tech Transformation Incubation Project (FDEKCXY08).

## Conflict of Interest

The authors declare that the research was conducted in the absence of any commercial or financial relationships that could be construed as a potential conflict of interest.

## Publisher's Note

All claims expressed in this article are solely those of the authors and do not necessarily represent those of their affiliated organizations, or those of the publisher, the editors and the reviewers. Any product that may be evaluated in this article, or claim that may be made by its manufacturer, is not guaranteed or endorsed by the publisher.
